# Role of Mitochondrial Iron Overload in Mediating Cell Death in H9c2 Cells

**DOI:** 10.3390/cells12010118

**Published:** 2022-12-28

**Authors:** Eddie Tam, Hye Kyoung Sung, Nhat Hung Lam, Sally You, Sungji Cho, Saher M. Ahmed, Ali A. Abdul-Sater, Gary Sweeney

**Affiliations:** 1 Department of Biology, York University, Toronto, ON M3J 1P3, Canada; 2 Muscle Health Research Centre, School of Kinesiology and Health Science, York University, Toronto, ON M3J 1P3, Canada

**Keywords:** iron overload, cell death, reactive oxygen species, mitochondria, MitoNEET

## Abstract

Iron overload (IO) is associated with cardiovascular diseases, including heart failure. Our study’s aim was to examine the mechanism by which IO triggers cell death in H9c2 cells. IO caused accumulation of intracellular and mitochondrial iron as shown by the use of iron-binding fluorescent reporters, FerroOrange and MitoFerroFluor. Expression of cytosolic and mitochondrial isoforms of Ferritin was also induced by IO. IO-induced iron accumulation and cellular ROS was rapid and temporally linked. ROS accumulation was detected in the cytosol and mitochondrial compartments with CellROX, DCF-DA and MitoSOX fluorescent dyes and partly reversed by the general antioxidant N-acetyl cysteine or the mitochondrial antioxidant SkQ1. Antioxidants also reduced the downstream activation of apoptosis and lytic cell death quantified by Caspase 3 cleavage/activation, mitochondrial Cytochrome c release, Annexin V/Propidium iodide staining and LDH release of IO-treated cells. Finally, overexpression of MitoNEET, an outer mitochondrial membrane protein involved in the transfer of Fe-S clusters between mitochondrial and cytosol, was observed to lower iron and ROS accumulation in the mitochondria. These alterations were correlated with reduced IO-induced cell death by apoptosis in MitoNEET-overexpressing cells. In conclusion, IO mediates H9c2 cell death by causing mitochondrial iron accumulation and subsequent general and mitochondrial ROS upregulation.

## 1. Introduction

Iron is a vital micronutrient: it plays central roles as a redox co-factor in oxidative phosphorylation and oxygen carrier in Heme groups of haemoglobin and myoglobin [1]. It is obtained primarily through dietary sources and its whole-body and cellular homeostasis is regulated at multiple levels [1,2,3]. During digestion, its absorption in the intestine, is regulated by divalent metal transport 1 (DMT1) (for non-heme iron) and heme carrier protein [3]. Once it has entered the body, it either binds to transferrin for transport in the serum or exists as non-transferrin-bound iron (NTBI) [2]. Transferrin-bound iron is taken up into endosomes by transferrin receptor 1 on the cell membrane and released into the cytoplasm through DMT1. Several cell types, including liver and pancreas, also express ferroportin to export iron out the cell [2,3].

Iron overload (IO) can be the direct result of hereditary haemochromatosis, or secondary to frequent transfusions or high dietary intake [1,2]. Abnormal iron metabolism has been associated with many diseases—including neurodegeneration, cancer, type 2 diabetes, and cardiovascular diseases [4,5,6,7]. Importantly, both iron deficiency and iron overload have been implicated in cardiomyopathy [8,9]. IO-related cardiomyopathy has become an important, yet underestimated cause of morbidity and mortality [9].

Iron is a transition metal that interconverts readily between ferrous (Fe^2+^) and ferric (Fe^3+^) forms and has potent pro-oxidant properties that play an important role in its biological activity [1]. Under IO, however, the excess unregulated labile NTBI can accumulate inside the cells, especially in the heart, where it promotes oxidative stress and increases production of reactive oxygen species (ROS) that go on to cause cellular damage [9], including mitochondrial dysfunction and lipid peroxidation [10].

MitoNEET (or *Cisd1*, Gene ID: 294362) is an iron–sulfur-containing protein residing in the mitochondrial outer membrane (OMM) [11]. MitoNEET regulates mitochondrial iron homeostasis and in this regard, its ability to transfer Fe-S clusters out of the mitochondria to other acceptor proteins may be one way by which it maintains mitochondrial iron levels [11]. Originally identified in adipocytes, MitoNEET overexpression in adipose or liver, reduced iron levels in the mitochondrial matrix, lowering ETC activity, fatty acid oxidation and consequently decreasing ROS production, thereby imparting metabolic benefits [12]. By contrast, reduction in MitoNEET expression, with a systemic inducible shRNA-mediated knockdown of MitoNEET, increased mitochondrial labile iron, ROS production, oxidative stress and insulin resistance [12].

The role of mitochondria in cell death is well-established. First, they contain Cytochrome c, which is released into the cytoplasm during apoptotic signalling, a key intersection of the intrinsic cell death pathway [13]. Furthermore, these organelles perform oxidative phosphorylation to provide the cells with ATP. They are present in abundance in metabolically active cells that rely on mitochondrial respiration, cardiomyocytes being the most salient example [14]. While iron is essential to mitochondrial function, being a component of the iron–sulfur clusters in Complexes I, II and III in the electron transport chain (ETC) [15], IO has been shown to disrupt this very process, resulting in overproduction and leakage of ROS (mainly superoxides) into the cytoplasm [10]. Mitochondrial ROS contribute significantly to IO-induced oxidative stress and subsequent cell damage or death [10]. Therefore, identifying mechanisms to limit the effects of IO on mitochondria may prove to mitigate the adverse cellular consequences of oxidative damage stemming from these organelles.

We hypothesize oxidative stress, specifically mitochondrial ROS, is a primary mechanism through which IO causes cell death in H9c2 cells. We show that MitoNEET overexpression in H9c2 protects against IO-induced cell death by reducing iron content in the mitochondria and preventing mitochondrial ROS.

## 2. Materials and Methods

### 2.1. Reagents

FeSO_4_ (Ferrous Sulfate Heptahydrate, 215422), N-acetyl-L-cysteine (NAC, A7250) and DCF-DA (2′,7′-dicholorodihydrofluorescein diacetate dye, D6883), Ampicillin (A9518) LB broth (L3522), G418 (A1720) were from MilliporeSigma (Burlington, ON, Canada). Hoechst nuclear dye (H10325), CellROX^TM^ Deep Red Reagent (C10422), Image-IT^TM^ DEAD Green^TM^ Viability Stain (I10291), MitoSOX^TM^ Red (M36008), MitoTracker^TM^ Deep Red FM (M22426) and CellEvent Caspase 3/7 Green Detection Reagent (C10423), Subcloning Efficiency^TM^, ReadyProbes^TM^ Cell Viability Imaging Kit (R37609), DH5α Competent Cells (18265017), Pierce Protease and Phosphatase Inhibitor Mini Tablets (A32959) were from Thermo Fisher (Waltham, MA, USA). Gibco brand Dulbecco’s Modified Eagle’s Medium (DMEM), fetal bovine serum (FBS) and 1% penicillin/ streptomycin were from Thermo Fisher (Waltham, MA, USA). 10-(6’-plastoquinonyl decyltriphenylphosphonium (SkQ1, HY-100474) was from MedChemExpress (Monmouth Junction, NJ, USA). FerroOrange was from Dojindo Molecular Technologies (Rockville, MD, USA). Anti-TOM20 antibody (sc-17764) and anti-Cytochrome c antibody (sc-25304) were purchased from Santa Cruz Biotechnology (Dallas, TX, USA). The Cytoscan^TM^-LDH Cytotoxicity Assay kit (786-210) was purchased from G-Biosciences (St. Louis, MO, USA). PBS containing calcium and magnesium was from Wisent Bioproducts (311-011-CL, Wisent Inc., St-Bruno, QC, Canada). BSA (ALB001, Bioshop, Burlington, ON, Canada).

### 2.2. Cell Culture

H9c2 rat embryonic cardiomyoblasts (ATCC, Manassas, VA, USA) were grown in DMEM supplemented with 10% FBS and 1% penicillin/ streptomycin (P/S) at 37 °C and a 5% CO_2_ atmosphere. The H9c2 cell line is a subclone from an original clonal cell line derived from embryonic BD1X rat heart tissue by Kimes and Brandt [16] and resemble immature embryonic cardiomyocytes [17]. Iron overload was induced by incubation of H9c2 in DMEM with 0.5% FBS and 1% P/S, supplemented with 100 µM FeSO_4_ for 24 h when cells reached approximately 70% of confluency. This concentration of FeSO_4_ is within the range typically used by researchers in the field to modulate the activity of iron regulatory proteins (IRP1 and IRP2) and trigger homeostatic processes to iron overload. NAC (500 nM) and SkQ1 (20 nM), a general antioxidant or mitochondrial specific antioxidant, respectively, were supplemented where indicated, 30 min prior to iron treatment and for the remainder of the 24 h. For assays requiring microscopy, cells were cultured on cover slips or imaging chambers at approximately 70% confluency for durations as specified in each assay.

### 2.3. Creation of Stable MitoNEET-Overexpressing Cell Line

*Escherichia coli* DH5α were transformed using heat shock with plasmids containing either the MitoNEET (pMitoN) gene or empty vector (EV) (Genescript, clone ID Ora10938 and SC1217, respectively). Cultured and transformed *E. coli* colonies were selected on an LB/ampicillin plate and further sub-cultured in LB/ampicillin media. pMitoN and EV plasmids were purified using the QIAGEN Plasmid Midi Kit and confirmed with visualization with ethidium bromide by agarose gel electrophoresis relative to a DNA molecular weight ladder (Abcam, Cambridge, UK). H9c2 cells at 70% confluency were then transfected with pMitoN and EV plasmids using the Lipofectamine 3000 transfection kit (Thermo Fisher, Waltham, MA, USA) for 48 h. Afterwards, transfected H9c2 cells were selected using 10% FBS DMEM containing 500 µg/mL G418 antibiotic for 5 days. Surviving cells were collected and sub-cultured in a 96-well plate at a low density in G418-containing DMEM + 10% FBS for 2 weeks. The 5 most confluent wells from the pMitoN plate were collected and characterized, whereas colonies from the EV plate were pooled and cultured. Following characterization, the colony lineage expressing the most pMitoN was selected for further expansion and use in this study. EV-transfected H9c2 cells were used as control.

### 2.4. Intracellular Iron Quantification

FerroOrange is a cell-permeant non-fluorescent dye in its unbound state but exhibits fluorogenic signal when bound to ferrous (Fe^2+^) ions. For live detection of intracellular iron, H9c2 were seeded at 70% confluence in DMEM + 0.5% FBS on µ-slide 8-chambered polymer coverslips (Ibidi, GmbH, Germany) and treated with or without FeSO_4_ (100 µM, for the indicated times) in the presence of FerroOrange (1 µM) and nuclei-staining dye Hoechst (100 nM) at 37 °C, 5% CO_2_. The cells were imaged every 5 min on a Nikon A1 Confocal Microscope fitted with an incubated chamber (37 °C, 5% CO_2_). Images were analyzed using NIS-Elements (Nikon).

### 2.5. Mitochondrial Iron Quantification

MitoFerroFluor, MFF (a kind gift from Dr John J. Lemasters), is a new fluorescent marker used for measuring mitochondrial iron [18]. MFF accumulates in the mitochondria where it exhibits a fluorescent signal that can be quenched by ferrous iron. For experimentation, H9c2 cells were seeded at 70% confluence in DMEM + 0.5% FBS on µ-slide 4-chambered polymer coverslips and treated with FeSO_4_ (100 µM, 24 h). At treatment endpoint, cells were washed with PBS three times, then incubated with MFF (2 µM) and Hoechst (100 nM) for 30 min in PBS at 37 °C protected from light. Cells were washed with PBS three times and imaged using Nikon A1 Confocal Microscope fitted with an incubated chamber (37 °C, 5% CO_2_). Mean MFF fluorescent intensity was quantified by ImageJ.

### 2.6. Reactive Oxygen Species Detection

CellROX Deep Red oxidative stress reagent is not fluorescent in a reduced state but exhibits a strong fluorescent signal when oxidized. For live detection of ROS production, H9c2 cells were seeded at 70% confluency in DMEM + 0.5% FBS on µ-slide 4-chambered polymer coverslips (Ibidi, GmbH, Germany), and pre-stained 15 min with Hoechst (100 nM) in DMEM + 0.5% FBS. At the start of imaging, CellROX Deep Red (2.5 µM) was added, alongside FeSO_4_ (100 μM), for the indicated times. Imaging was performed with 20× objective (Nikon A1 confocal microscope) at 5 min intervals in a 37 °C, 5% CO_2_ live-cell chamber. For fixed detection of ROS production, H9c2 cells were seeded onto glass coverslips and treated with FeSO_4_ (100 μM, 4 h). CellROX^®^ Deep Red (2.5 µM) was added to each well for 15 min. Cells were washed briefly with PBS and fixed with 10% formalin, before being mounted onto to a glass slide with VECTASHIELD mounting medium plus DAPI. Images were taken at 40× objective (Nikon A1). Images were analyzed using NIS-Elements (Nikon). When using DCF-DA, cells were washed with warmed PBS following FeSO_4_ treatment (100 μM, 4 h), and incubated with DCF-DA (20 µM) in PBS for 30 min at 37 °C protected from light. Afterwards, the cells were washed 3 times with warmed PBS. Fluorescence from DCF-DA was measured at 490 nm excitation and 520 nm emission using a Varioskan LUX plate reader (Thermo Fisher, Waltham, MA, USA). The antioxidants NAC and SkQ1 were used, as indicated.

### 2.7. Mitochondrial Reactive Oxygen Species Detection

MitoSOX Red is selectively targeted to mitochondria and exhibits a strong fluorescent signal upon oxidation. For live detection of mitochondrial ROS production, H9c2 cells were seeded at 70% confluency in DMEM + 0.5% FBS on a µ-slide 4-chambered polymer coverslip and treated with FeSO_4_ (100 μM, 4 h). At treatment endpoint, cells were washed with PBS three times, then incubated with MitoSOX Red (5 µM) and Hoechst (100 nM) for 30 min at 37 °C protected from light. Cells were washed times with PBS then imaged using a Nikon A1 confocal microscope, fitted with an incubated chamber (37 °C, 5% CO_2_). Mean MitoSOX intensity was quantified by ImageJ.

### 2.8. Caspase 3/7 Detection Kit

CellEvent Caspase 3/7 Detection Reagent is cell-permeant reagent and consists of the peptide (DEVD) conjugated to a nucleic acid-binding dye. Upon cleavage of the DEVD recognition sequence by caspases 3/7, the dye is released to bind cellular DNA increasing its fluorescent signal. For detection, H9c2 cells were plated at 70% confluency in 96-well plates (non-tissue culture coated) and treated with FeSO_4_ (100 µM, 24 h) with or without co-treatment with antioxidants NAC (500 nM) and SkQ1 (20 nM). At the treatment endpoint, cells were incubated with Caspase 3/7 Detection Reagent (10 µM) and Hoechst (100 nM) for 30 min at 37 °C protected from light. Afterward, images were obtained and analyzed to characterize cells with activated Caspase 3/7 pathways through the CellInsight CX7 High Content Screening platform (Thermo Fisher, Waltham, MA, USA), Absorption/Emission: 511/533 nm.

### 2.9. Flow Cytometry

Alexa Fluor^®^ 488 Annexin V/Dead Cell Apoptosis Kit (V13241, Thermo Fisher, Waltham, MA, USA) was used to detect the early and late stages of apoptosis using annexin V and propidium iodide (PI). Annexin V detects phosphatidyl serine on the surface of the cell membrane surface during early stages of apoptosis. PI is a membrane impermeable nucleic acid binding dye used to detect late apoptotic cells, as per manufacturer’s instructions. H9c2 cells were plated on 6-well plates and cultured to reach 70–80% confluency. Treatment conditions for cells are outlined in the figure legends-conditions included 0.5% FBS media, FeSO_4_ 100 µM, and co-treatment with NAC 500 nM or 20 nM SkQ1. Following treatments, cells were lifted off the culture dishes with trypsin-EDTA in PBS and immediately incubated with annexin V and PI diluted in 1x annexin-binding buffer (30 min at 4 °C), then mixed with additional 1x annexin-binding buffer. Cells were placed on ice until immediate flow cytometry analysis using the AttuneTM NxT Acoustic Focusing cytometer (Thermo Fisher, Waltham, MA, USA) with the BL1 and BL3 lasers for Annexin V and PI, respectively.

Cells were stained with MitoSOX Red assay and monobromobimane (mBBr) at treatment endpoints as per the manufacturer’s protocol. Following staining, cells were washed and lifted off the culture dishes with trypsin-EDTA in PBS and suspended in 2% FACS buffer on ice until immediate flow cytometry analysis on the Attune^TM^ NxT Acoustic Focusing cytometer (Invitrogen, Waltham, MA). The VL2 and BL1 lasers were used for mBBr and MitoSOX Red, respectively. Flow cytometry data were analyzed using FlowJo^®^ software (Tree Star Inc., Ashland, OR, USA).

### 2.10. Immunocytochemistry

H9c2 cells were grown on glass coverslips in 24-well plates. They were allowed to reach 70% confluency and treated with FeSO_4_ (100 µM, 24 h) in DMEM + 0.5% FBS without or without the co-treatment of antioxidants 500 nM NAC and 20 nM SkQ1. At the treatment endpoint, the coverslips were washed briefly with PBS (PBS with added Mg^2+^ and Ca^2+^) and fixed with 10% formalin solution for 30 min. Following 3 more washes, the cells were permeabilized using PBS containing 0.5% Triton-X detergent for 30 min and blocked with PBS containing 3% BSA for 1 h. Afterwards, the cells were incubated in 3% BSA in PBS solution containing mouse anti-TOM20 (Santa Cruz, for mitochondria detection) and rabbit anti-Cytochrome c (Abcam, Cambridge, UK) at 1:500 dilution overnight at 4 °C. Cells were then washed 3 times with PBS containing 0.5% Tween-20 and incubated in 3% BSA in PBS solution containing anti-rabbit IgG Alexa Fluor 555 and anti-mouse IgG Alexa Fluor 488 (Thermo Fisher, Waltham, MA, USA) at 1:500 dilution for 1 h at room temperature. Afterwards, cells were washed 3 times with PBS containing 0.5% Tween-20. Coverslips were mounted onto slides in mounting medium containing a 1:1 mixture of VECTASHIELD anti-fade mounting medium with DAPI and imaged using the Nikon A1 Confocal Microscope. Images were analyzed using NIS-Elements (Nikon). For 3D images, z-stacks were captured, then rendered on IMARIS 9.9.0 using the surfaces tool.

### 2.11. Cell Death Assays

Cell rupture was quantitatively evaluated by measuring the released lactate dehydrogenase (LDH) with CytoScan^TM^ LDH Cytotoxicity Assay (G-Biosciences, 786-210). H9c2 cells were treated with FeSO_4_ (100 μM, 24 h) in DMEM + 0.5% FBS with or without antioxidants NAC (500 nM) and SkQ1 (20 nM). The cultured media were mixed in 1:1 ratio with the mixed substrate solution followed by incubation for 30 min in the cell culture incubator (37 °C, 5% CO_2_) protected from light. The assay was quantified by absorbance at 490 nm subtracted by 680 nm and plotted as a relative value over the control condition.

Image-IT™ DEAD Green™ Viability Stain was used to detect dead cells with compromised plasma membrane. H9c2 cells were plated at 70% confluency in DMEM + 0.5% FBS in 96-well plates (non-tissue culture coated) and treated with FeSO_4_ (100 μM, 24 h) with or without co-treatment with antioxidants NAC (500 nM) and SkQ1 (20 nM) for. At treatment endpoint, the Viability Stain (100 nM) and Hoechst (100 nM) were added to the cell media for 30 min (37 °C, 5% CO_2_) protected from light. Afterwards, treatment media were removed, and cells were washed briefly with PBS and fixed with 10% formalin solution. The images were obtained and analyzed to characterize dead compromised cells through the CellInsight CX7 High Content Screening platform in the FITC (green) channel.

ReadyProbes™ Cell Viability Imaging kit was used to determine viability of cells. H9c2 cells were seeded at 70% confluence in 24-well plates and treated with FeSO_4_ (100 μM, 24 h) in DMEM + 0.5% FBS. At treatment endpoint, cells were stained according to manufacturer’s protocol, then imaged using EVOS FL Auto 2 Imaging system (Thermo Fisher, Waltham, MA, USA).

### 2.12. Quantitative Polymerase Chain Reaction (qPCR)

H9c2 cells were plated on a 6-well plate. When the confluency reached to 70%, cells were treated with FeSO_4_ (100 μM, 24 h) in DMEM + 0.5% FBS. At the treatment endpoint, cells were trypsinized and washed 3 times with PBS at 4 °C. Cells were then lysed and processed to collect RNA by using the RNeasy Mini Kit (QIAGEN). The extracted RNA was quantified using the plate reader-based µDrop^TM^ Plate (Thermo Fisher, Waltham, MA, USA) and approximate 400 ng of total RNA per sample was used in reverse transcription using the RevertAid RT Reverse Transcription Kit (Thermo Fisher) using random hexamer primer to obtain total cDNA. The qPCR solution was prepared with cDNA, the iQ^TM^ SYBR^®^ Green Supermix (Bio Rad, Hercules, CA, USA), and the respective primers (outlined in Table 1) and the reaction was performed using a Bio Rad qPCR machine. The samples were placed at 95 °C for enzyme activation, 95 °C for denaturation, 60 °C for annealing, 72 °C for extension, followed by a plate read. Data was quantified and analyzed using the delta-delta Ct method normalized over the 18S rRNA. 

### 2.13. Western Blot

Following treatment endpoint (100 μM FeSO_4_ in DMEM + 0.5% FBS for 24 h), H9c2 cells were lysed in RIPA lysis buffer containing a protease and phosphatase inhibitors. After denaturing lysates in Laemmli sample buffer for 10 min at 90 °C, samples were resolved on an SDS-PAGE gel and transferred onto PVDF membrane. The membranes were blocked with 3% BSA for 1 h at room temperature. Membranes were then incubated overnight at 4 °C in primary antibodies against: Cleaved caspase-3 (Asp175, 1:1000, Cell signaling, #9661), CISD1/MitoNEET (D5M4C, 1:1000, Cell signaling technology (CST), Danvers, MA, USA, #83775), GAPDH (14C10, 1:1000, CST, #2118). Membranes were then washed and incubated in the corresponding horse-radish peroxidase (HRP)-linked secondary antibody for 1 h at room temperature: anti-mouse IgG, HRP-linked antibody (1:10,000, CST, #7076) and anti-rabbit IgG, HRP-linked antibody (1:10,000, CST, #7074). The antibody-antigen complex was reacted with enhanced chemiluminescence (Bio Rad, Hercules, CA, USA) reagents before developing and the signal was detected by using CL XPosure Film (Thermo Fisher, Waltham, MA, USA). The density of each band was quantified using Image J, NIH Image.

### 2.14. Statistics

Data are given as mean ± SEM, and one-way analysis of variance was used for comparison in GraphPad Prism 8.0. All experiments were performed with a minimum of 3 biological replicates (n = 3), unless otherwise stated. Differences were significant at * *p* < 0.05, ** *p* < 0.01, *** *p* < 0.001 vs. Control. # *p* < 0.05, ## *p* < 0.01 vs. FeSO_4_.

## 3. Results

### 3.1. Intracellular IO Results in Increased Oxidative Stress in H9c2 Cells

**Figure 1:**
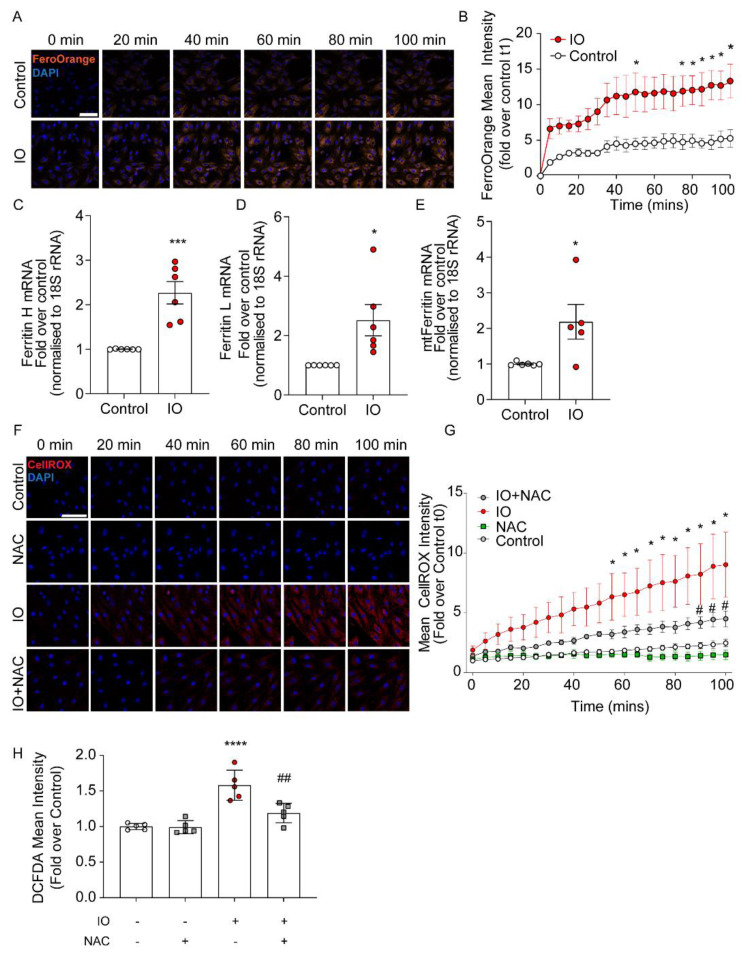
Characterization of intracellular iron and oxidative stress due to iron overload in H9c2. (**A**) Time-course of live-cell confocal imaging examining intracellular iron in H9c2 cells, using FerroOrange and Hoechst nuclear dye (blue). Cells were treated with 100 µM FeSO_4_ concurrently with FerroOrange dye (1 µM) and Hoechst (100 nM). (**B**) Fluorescence mean intensity of FerroOrange in IO or control was measured at 5 min intervals and calculated as the fold over Control at t1 (5 min time point) for both conditions (n = 4). (**C**–**E**) H9c2 were cultured in 6-well plates and cultured with 100 µM FeSO_4_, or not (Control), for 24 h. (**C**) Quantification of Ferritin Heavy and (**D**) Light chains mRNA (normalized to 18S rRNA) and (**E**) mito-Ferritin mRNA (normalized to 18S rRNA), presented as fold over Control (n = 6). (**F**) Time-course of live-cell confocal imaging examining ROS production under IO (100 µM FeSO_4_), using CellROX Deep Red (Red) and Hoechst to stain nuclei (blue). NAC (500 nM) was used as a ROS inhibitor added 30 min prior to the start of IO. (**G**) Fluorescence mean intensity of CellROX Deep Red was measured at 5 min intervals and calculated as the fold over control at t1 (5 min time point) for all conditions (n = 4). (**H**) Quantification of ROS production under IO (4-h) with DCF-DA ROS dye (see Methods). NAC was used as a ROS inhibitor added 30 min prior to IO treatment (n = 5). Scale bars in B and G denotes 50 µm. * *p* < 0.05, *** *p* < 0.001, **** *p* < 0.0001 relative to Control; # *p* < 0.05, ## *p* < 0.01 relative to IO.

We employed an in vitro model of IO in heart cells by incubating H9c2 cells with 100 μM FeSO_4_ in DMEM + 0.5% FBS for various times, up to 24 h. In Figure 1A, H9c2 cells were incubated for the indicated time course followed by iron detection with Fe^2+^-sensitive fluorescent dye FerroOrange, as described in the Methods. Iron accumulation steadily increased, reaching significance by 75 min, compared to cells just in media, trending upwards until the 100 min time point (Figure 1A,B). Note that later studies with a mitochondria-targeted iron sensor indicated significant mitochondrial iron accumulation. This will be further elaborated in Figure 4. IO induced increased expression of the iron storage proteins, Ferritin. The cytoplasmic (heavy chain, Ferritin H and light chain, Ferritin L) and mitochondrial (mtFerritin) isoforms were elevated more than 2-fold after 24 h of iron accumulation in the cytosol and likely the mitochondria (Figure 1C–E). Next, we examined the pro-oxidative effects of IO on H9c2 cells through live imaging using the fluorescent CellROX dye (see Methods). Here, the time course of iron uptake demonstrated that IO resulted in a significant increase in ROS production compared to control (no FeSO_4_ in the media) by the 55th min and continued to increase until the final 100th min of the treatment (Figure 1F). This increase was moderated when cells were pre-treated with the general antioxidant NAC, a glutathione precursor, and significantly lowered ROS accumulation by the 85th min of the 100 min time course (Figure 1G). These results of ROS generation induced by iron uptake and the moderation by NAC, were corroborated using the quantitative ROS-binding fluorescent dye DCF-DA, measured at a 4 h time point of iron uptake (Figure 1H).

### 3.2. Increased ROS Production Mediates Apoptosis in H9c2 Cells

**Figure 2:**
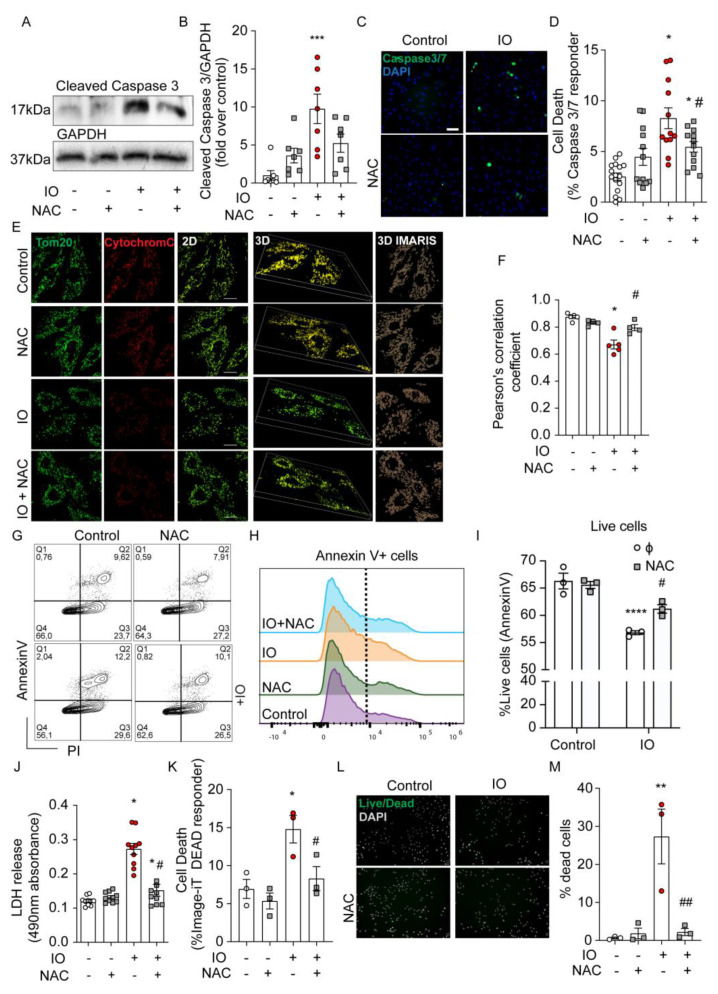
Iron overload activates apoptosis and increases H9c2 cell death. H9c2 cells were treated with FeSO_4_ (100 μM, 24 h) with or without NAC (500 nM, 24 h, with 30 min prior) co-treatment versus Control (DMEM + 0.5% FBS). (**A**) Western blot analysis of H9c2 lysates for the abundance for the apoptosis protein marker, cleaved-Caspase 3, in (**B**) Quantification of cleaved Caspase 3 band intensity (ImageJ, NIH Image) was normalized to GAPDH intensity for each condition and presented as fold over Control. (**C**) Representative CellInsight images from Caspase 3/7 Detection fluorescence assay to examine apoptotic cells with activated Caspase 3/7 cleavage (green nuclei) overlayed with all nuclei (marked by Hoechst). (**D**) Quantification of (**C**): Number of green positive nuclei as % of total nuclei in all cell wells (n = 4 experiments). (**E**) Examination of intrinsic apoptotic activation under IO by immunofluorescence: TOM20 (green) and Cytochrome C (red). Co-localization of cytochrome c (red) to the mitochondrial membrane (green). Representative images in 2D and 3D image are shown. For 3D, IMARIS software was used to render z-stack images (see Methods). (**F**) The TOM20 and Cytochrome C overlap was quantified in 25 cells per experiment by Pearson’s correlation coefficient for (n = 4–5, experiments). (**G**) Representative plots showing frequency of Annexin-V and/or propidium iodide (PI) labeled cells following treatment. (**H**,**I**) Graphs show the percentage of live cells (PI-negative) from three experiments like the one in panel G. (**J**) LDH release detection assay for cell rupture, quantified as 490 nm absorbance (n = 4–5 experiments, in duplicate). (**K**) Quantification of apoptotic cells with compromised plasma membrane integrity detected by Image-iT DEAD kit, presented as % of total nuclei. (**L**) Assessment of cell viability using ReadyProbes Cell Viability kit. (**M**) Quantification shows the number of dead cells as a % of total cells. Pre-treatment with NAC was used, where indicated. The scale bars shown, denotes 50 µm in C and I, and 20 µm in E. * *p* < 0.05, ** *p* < 0.01, *** *p* < 0.001, **** *p* < 0.0001 relative to Control; # *p* < 0.05, ## *p* < 0.01 relative to IO.

Next, we investigated the role ROS production plays in the mediation of H9c2 cell death. Cleavage of Caspase 3 is a key intersection point of apoptotic pathways. Here, we demonstrated (by Western blot) that IO resulted in an elevated amount of cleaved caspase 3, relative to control (Figure 2A,B) and this effect was reduced when cells were pre-treated with NAC (Figure 2A,B). Using a complementary technique, we examined Caspase 3/7 activation with a fluorescence-based assay (See Methods) (Figure 2C). The quantification of the percent of positive cells for Caspase 3/7 activation was highly reflective of the Western blot results in Figure 2A. Another indicator of apoptosis is the release of Cytochrome c from mitochondria (Figure 2E,F). 3D-imaging with IMARIS software reveals Cytochrome 3 localized to the mitochondria, as demarcated by TOM20 (Figure 2E), but the reduction in TOM20 and Cytochrome C overlap by the IO treatment, suggested Cytochrome C loss to the cytosol in the process of apoptosis. NAC reversed Cytochrome C loss, implicating the reduction of cellular ROS slows H9c2 apoptosis induced by IO (Figure 2F). Flow cytometry using Annexin V/PI staining revealed a significant decrease in live cell numbers (increased apoptotic cells) by IO, relative to control, that was modestly spared by NAC antioxidant co-treatment (Figure 2G–I). IO at least doubled the cell rupture events measured by LDH release and this was almost completely spared by NAC co-treatment (Figure 2J). Measurement of loss of plasma membrane (PM) integrity with the Image-iT DEAD kit is complementary to LDH release, and this is reflected in the ~2-fold increase in loss of PM integrity by IO and the near full recovery by NAC co-treatment (Figure 2K). Lastly, analysis of cell death using the ReadyProbes Cell Viability kit according to the manufacturer, suggested a ~10-fold increase in dead cells by IO that was completely attenuated by NAC co-treatment, similar to LDH release and Image-iT DEAD assay findings (Figure 2L,M). Together, IO induced an apoptotic response in H9c2 (cleaved caspases, Cytochrome C loss from mitochondria, Annexin-V binding to cell surface membranes), which could be attenuated by the antioxidant NAC. Left unchecked, 24 h of IO significantly triggered cell death by several measures of loss of cell membrane integrity. Protection by NAC suggests ROS-mediated activation of the apoptosis program by IO.

### 3.3. IO-Induced Mitochondrial ROS Is an Important Mediator of Apoptosis in H9c2 Cells

**Figure 3:**
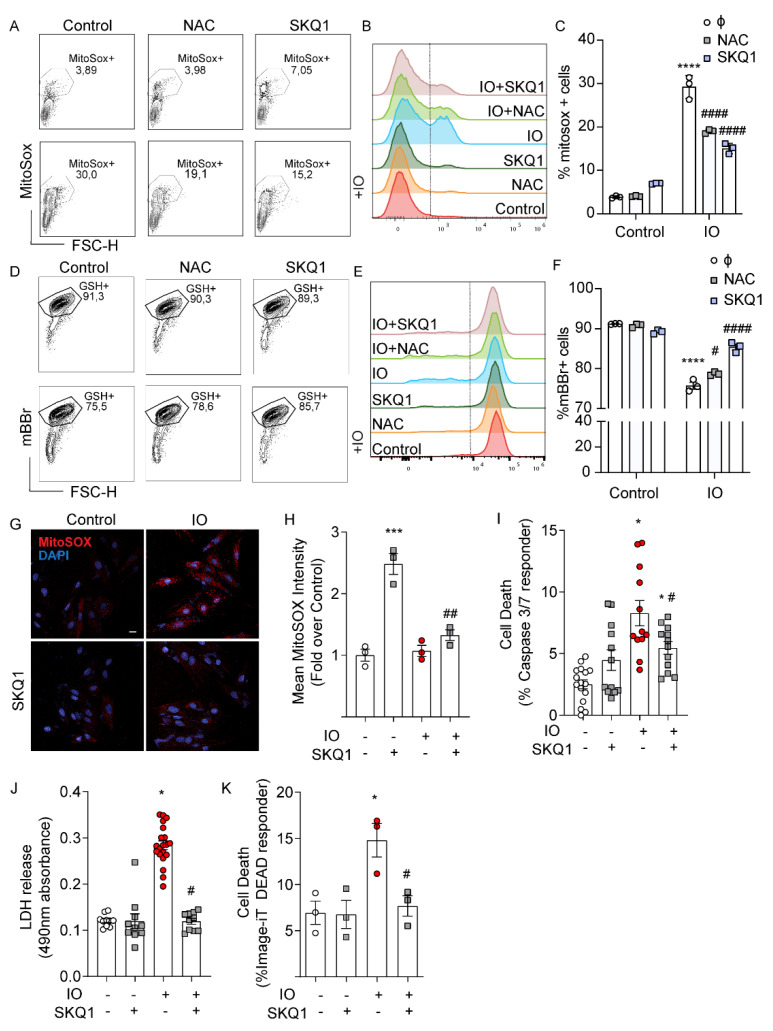
Mitochondrial ROS contributes to cell death induced by iron-overload. H9c2 cells were treated with FeSO_4_ (100 μM, 24 h) with or without SkQ1 (20 nM, 24 h, plus 30 min prior) co-treatment versus Control (DMEM + 0.5% FBS). (**A**–**C**) Flow cytometry analysis with MitoSOX Red. (**A**) Flow cytometry gating for MitoSOX-positive cells. (**B**) Overlayed histograms showing the mean fluorescence intensity (MFI): right of the line are positive cells for increased mitochondrial ROS (**C**) Graphical representation of MitoSOX-positive cells. (**D**) Flow cytometry gating for mBBR-labeled cells (GSH+). (**E**) Overlayed histograms showing the mean fluorescence intensity (MFI), right of the line are cells positive for mBBR-GSH interaction. (**F**) Graphical representation of mBBR-positive cells from three experiments like that shown in panel D. (**G**) Analysis of mitochondrial ROS by confocal analysis using MitoSOX Red staining. (**H**) Quantification of mean puncta intensity of MitoSOX Red staining (n = 3). (**I**) Quantification of mean puncta intensity for Caspase 3/7 cleavage as % of total nuclei, using the CellEvent Caspase 3/7 Detection kit (n = 4). (**J**) LDH release assay and (**K**) Image-iT DEAD assay examining the effect of SkQ1 on IO-induced cell death (n = 4). Scale bar in G denotes 20 µm. * *p* < 0.05, *** *p* < 0.001, **** *p* < 0.0001 relative to Control; # *p* < 0.05, ## *p* < 0.01, #### *p* < 0.0001, relative to IO.

Based on the evidence of appreciable mitochondrial accumulation of iron suggested by mtFerritin expression after 24 h of IO (Figure 1C), we examined if IO induced mitochondrial ROS overproduction. MitoSOX Red is a fluorescent dye that is targeted to the mitochondria and is used to detect mitochondrial ROS. Flow cytometry analysis of MitoSOX Red demonstrated that IO induced a significant upregulation of mitochondrial ROS (Figure 3A–C). Interestingly, co-treatment with SkQ1, a cell-permeant and mitochondrial-specific antioxidant [19], prevented the upregulation of mitochondrial ROS by IO just as well as NAC, if not better (Figure 3A–C). mBBr is a fluorescent probe that binds to glutathione, interacting with cells in an overall reduced state, compared to an oxidized state. Based on mBBr staining of H9c2 cells, IO moderately shifted cells towards a more oxidized state (Figure 3D–F) and this was slightly reversed by NAC and almost completely reversed by SkQ1. These results were visually reinforced using a confocal microscopy approach for MitoSOX Red staining (Figure 3G,H). MitoSOX Red staining was especially punctate in the IO condition, consistent with its affinity for mitochondria, and SkQ1 reduced MitoSOX Red staining by greater than 50% (Figure 3G,H). Apoptosis analyses with the Caspase 3/7 fluorescent reporter illustrated SkQ1 was completely effective in reducing apoptosis signaling in IO-treated cells (Figure 3I), PM integrity were assessed using LDH release and Image-iT DEAD assays (Figure 3J,K). As in Figure 2, IO triggered a > 2-fold increase in lytic cell death, but here we observed that SkQ1 treatment completely attenuated IO-induced cell death (Figure 3J,K) that was complementary to NAC result (Figure 2J,K). These data implied that mitochondrial ROS plays an important role in mediating cell death of H9c2 cells under IO.

### 3.4. MitoNEET Overexpression Reduces Mitochondrial Iron Content and IO-Induced Oxidative Stress

**Figure 4:**
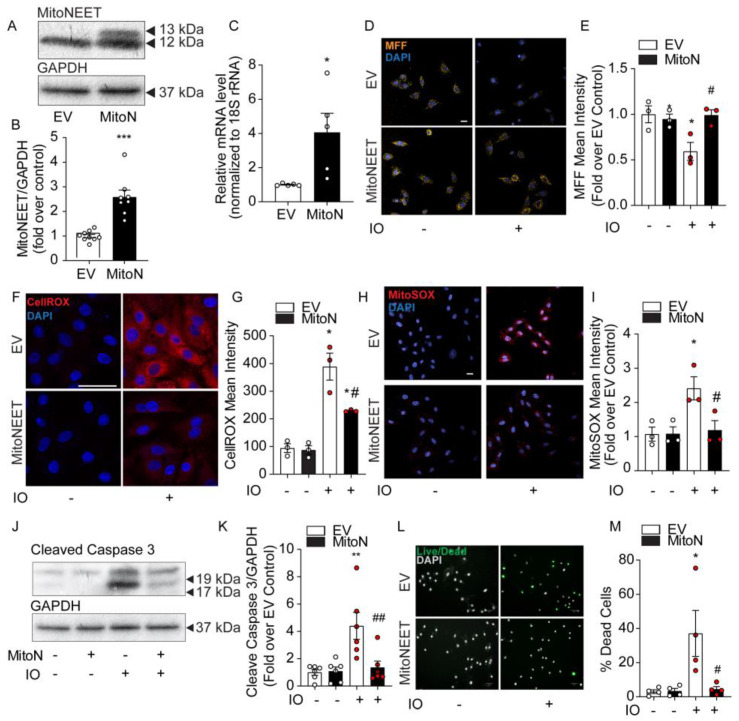
MitoNEET overexpression lowers cellular and mitochondrial ROS and protects H9c2 cell viability under conditions of iron overload. Characterization of MitoNEET overexpression in H9c2 cells. Control cells (EV) and MitoNEET overexpressing cells (MitoN) were treated with FeSO_4_ (100 μM, 24 h). (**A**) Western blotting for MitoNEET in EV and MitoN cells showed expression of the exogenous flagged-tagged MitoNEET (13 kDa) relative to the endogenous version (12 kDa). (**B**) Densitometric quantification of total MitoNEET expression relative to GAPDH using ImageJ software. (**C**) MitoNEET mRNA quantification (relative to 18S rRNA) by qPCR in EV and MitoN cells (n = 5). (**D**) Confocal images of mitochondrial MFF fluorescent dye in EV and MitoN cells under IO is quenched in proportion to labile mitochondrial iron levels. (**E**) Quantification of MFF pixel intensity (n = 3). (**F**) Analysis of ROS production in EV and MitoN cells under IO, as detected by CellROX Deep Red. (**G**) Quantification of CellROX Deep Red pixel intensity (n = 3). (**H**) Analysis of mitochondrial ROS by confocal analysis using MitoSOX Red. (**I**) Quantification of MitoSOX Red pixel intensity (n = 3). (**J**) Western blot analysis of the apoptotic protein biomarker, cleaved-Caspase 3, under IO in EV and MitoN cells. (**K**) Densitometric quantification of total MitoNEET expression relative to GAPDH using ImageJ software (n = 7). (**L**) Assessment of cell viability with ReadyProbes Cell Viability Imaging Kit. (**M**) Quantification of ReadyProbes positive cells is expressed as a % of total cells (n = 4). Scale bar denotes 20µm for D, H, and F, and 50 µm for L. * *p* < 0.05, ** *p* < 0.01, *** *p* < 0.001 relative to EV Control; # *p* < 0.05, ## *p* < 0.01, relative to EV + IO.

To further investigate the role of mitochondrial iron in the dysregulation of ROS production and cell death under IO, we created a H9c2 cell line stably overexpressing MitoNEET. By its activity of binding and releasing Fe-S clusters and its location on the outer mitochondrial membrane, it is thought to be involved in shuttling iron out of or into the mitochondrion) [11,12]. Stable overexpression was verified by Western blot. The transfected MitoNEET is roughly 1 kDa (13 kDa) heavier than the endogenous form (12 kDa), due to the presence of a FLAG tag (Figure 4A). Densitometric quantification of both bands indicated total MitoNEET overexpression was approximately 2.5-fold relative to endogenous expression (EV, Figure 4B). Quantitative PCR indicated the total mRNA expression of MitoNEET was elevated nearly 4-fold (Figure 4C).

Next, we examined the levels of mitochondrial iron content using MFF, a fluorescent probe that selectively accumulates in the mitochondria where it exhibits a fluorescent signal that is quenched by free iron [18]. In Figure 4D, MFF staining is punctate in a pattern reminiscent of mitochondria’s intracellular distribution. IO was observed to significantly quench MFF fluorescence in EV Control cells, but not in the MitoNEET-overexpressing cell line, indicating the ability of MitoNEET to reduce mitochondrial iron content (Figure 4D,E). The consequence of MitoNEET preserving normal levels of mitochondrial iron were marked. CellROX measurement of cellular ROS was increased nearly 4-fold in EV-Control cells with IO, but this decreased by 50% with MitoNEET overexpression (Figure 4F,G). Importantly, MitoSOX Red revealed mitochondrial ROS was increased by ~2.5-fold by IO in EV-Control cells, but was completely normalized back to control levels, by MitoNEET overexpression, compared to cells that did not receive iron-loading, (Figure 4H,I) These results clearly demonstrate the ability of MitoNEET overexpression to protect the cells from iron-induced increases in cellular and mitochondrial ROS production. These findings correlated with the protection against apoptosis activation and cell death. Upon IO, MitoNEET-overexpressing cells exhibited much lower levels of cleaved-caspase 3 by Western blotting (Figure 4J,K), suggesting a quieting of apoptosis. In addition, assessment of cell viability using ReadyProbes Cell Viability kit indicated IO could trigger an ~8-fold increase in cell death that was entirely prevented in cells overexpressing MitoNEET (Figure 4L,M). Overall, these data demonstrated the protective effect of MitoNEET overexpression in inhibiting mitochondrial iron accumulation, subsequent oxidative stress (increase ROS) and ultimately, preventing cell death caused by IO.

## 4. Discussion

Cardiomyopathy and heart failure are common pathologic endpoints for many patients with metabolic syndrome [20]. Such outcomes have been thought to have multi-factorial causes, including hypertension, insulin resistance, inflammation, and cardiomyocyte death [21]. IO-related cardiomyopathy and heart failure have been a primary focus of studies examining genetic traits that either perturb iron metabolism or medical conditions that require therapeutic transfusions [1,2]. In the last decade, however, there has been increasing interest in the intersection between IO pathologies and the metabolic syndrome, since serum ferritin levels (which can be used as a marker of IO) have been strongly associated with the severity of insulin resistance and metabolic syndrome [19,20] —sparking a re-emergence of the terminology “dysmetabolic iron overload syndrome (DIOS)” [22,23]. Recent evidence pinpoints the heart as a key organ affected in these concurrent diseases due to heightened oxidative stress and insulin resistance [24].

In our current study, we examined cell death by IO-induced oxidative stress in H9c2 cells cultured with FeSO_4_ for up to 24 h to simulate an acute model of IO. Using FerroOrange staining we observed significant intracellular iron accumulation within 2 h of treatment time, that was paralleled by significantly increased cellular ROS levels within the same time frame (tracked by CellROX or DCF-DA) and this could be moderated by the general antioxidant NAC. Longer incubation times with FeSO_4_ upregulated expression of the cytoplasmic and mitochondrial isoforms of the iron-storing proteins, Ferritin. Heightened ROS was shown to trigger the apoptosis program (Cleaved-caspase 3/7, Cytochrome C loss from mitochondria, increased PM phosphatidylserine detection with Annexin V) and increased the incidence of lytic cell death (e.g., LDH release) within the 24 h time-frame studied. Having observed that iron accumulation upregulated mitochondrial Ferritin, and the MFF detection of iron elevation within the mitochondria, the role of mitochondria-specific ROS generation was examined. Using MitoSOX, we observed increased production of mitochondrial ROS in IO, which was prevented by treatment with the mitochondrial antioxidant, SkQ1. IO-induced mitochondrial ROS induced apoptosis and cell death which were also reduced or prevented by SkQ1, thus implicating mitochondrial ROS as a causative factor.

MitoNEET is a recently discovered mitochondrial protein [25,26,27], originally characterized by its interaction with pioglitazone [28]. While there has been ample literature studying MitoNEET’s function, related to its binding of iron–sulfur complexes and its similarities to other (NEET) proteins containing CDGSH domains [27], studies investigating its roles in biological systems, especially those employing gain- or loss-of-function genetic alterations, are only just emerging [12,29]. It was from these studies that MitoNEET was found to regulate the mitochondrial respiration rate when overexpressed, likely by reducing the iron levels in the mitochondrial matrix [12]. Based on this evidence, we created a H9c2 cell line, stably overexpressing MitoNEET to examine how it may partition intracellular iron content. Under IO treatment, we found that these cells accumulated less iron in their mitochondria using the mitochondrial iron-binding fluorescent dye, MFF [18]. This observation correlated with less cellular and mitochondrial ROS overproduction and reduced biomarkers of apoptosis-related cell death and reduced cell lysis. These results established the potential of MitoNEET as a safeguard against IO at the cellular and mitochondrial levels.

IO-induced oxidative stress is well characterized as an important contributor to the pathogenesis of IO cardiomyopathy, myocardial injuries and heart failure [30,31]. From our current model, events underpinning IO cardiomyopathy could be attributed to IO within the mitochondria. By inhibiting the effects of mitochondrial iron accumulation (increased mitochondrial ROS) with the SkQ1 antioxidant or iron accumulation within this organelle, with MitoNEET expression, the deleterious effects of iron overload could be mitigated.

Regulation of mitochondrial iron by MitoNEET’s has not been fully elucidated. The transfer of its iron–sulfur clusters to acceptor proteins can be dependent on its own oxidative status. Specifically, in an oxidized form, this transfer occurs rapidly while it is inhibited when MitoNEET is reduced [32]. MitoNEET itself is reducible by biological antioxidants such as glutathione [27,33]. MitoNEET also exhibits lower iron–sulfur transfer kinetics when interacting with the anti-diabetic drugs in the thiazolidinedione class, such as pioglitazone, as these compounds stabilize MitoNEET structure and its binding affinity to the iron–sulfur clusters [32]. In a separate study of a spinal cord injury model, this interaction was shown to be essential in pioglitazone’s therapeutic effects [33]. Given that pioglitazone is insulin-sensitizing and iron overload has been shown to result in insulin resistance in the heart [24], pioglitazone’s interaction with MitoNEET and potential involvement in mitochondrial iron regulation may be a basis of part of its therapeutic impact.

Our study is limited by utilizing an in vitro model of iron overload in H9c2 cells to examine its consequences on cell viability. However, with a rising prevalence of metabolic syndrome and diabetes worldwide, secondary IO and the associated DIOS could be significant risk factors of cardiomyopathy, heart failure, and death [34]. Thus, our study points to prevention or therapeutic intervention against IO may prove advantageous in treating these maladies. Our study showed the beneficial role played by MitoNEET in counteracting IO and its consequences at the cellular level. While MitoNEET overexpression may not be clinically feasible, therapies utilizing antioxidants and thiazolidinediones may enhance MitoNEET activity and is worthy of further investigation.

## Figures and Tables

**Table 1:** Primer sequences used for qPCR.

Gene	Primer	Sequence (5′-3′)
18S rRNA	Forward	CCATAAACGATGCCGACTG
	Reverse	CGCTCCACCAACTAAGAAC
Ferritin Heavy Chain	Forward	CTTTGCAACTTCGTCGCTCC
	Reverse	AGTCATCACGGTCAGGTTTCTTT
Ferritin Light Chain	Forward	AGACCCTCACCTCTGTGACT
	Reverse	GGCGGTTACAAAGCTGCCTA
Mito-Ferritin	Forward	TCCTGGACTTGCATACTCTGGCCTCAG
	Reverse	GCTTGTCGAAAAGATACTCCGCTAGG
MitoNEET	Forward	AAGACAAACCCGAAGGTGGTG
	Reverse	TCGCAGAAGGGGAACTTTTT

## Data Availability

Data available upon reasonable request.
